# Effect of Pt doping on the preferred orientation enhancement in FeCo/SiO_2_ nanocomposite films

**DOI:** 10.1038/s41598-019-47215-3

**Published:** 2019-07-23

**Authors:** Mei Liu, Linglong Hu, Yue Ma, Ming Feng, Shichong Xu, Haibo Li

**Affiliations:** 1 Key Laboratory of Functional Materials Physics and Chemistry Ministry of Education, Jilin Normal University, Changchun, 130103 China; 2 Key Laboratory of Preparation and Application of Environmental Friendly Materials of the Ministry of Education, Jilin Normal University, Changchun, 130103 China; 3 National Demonstration Center for Experimental Physics Education, Jilin Normal University, Siping, 136000 China

**Keywords:** Synthesis and processing, Magnetic materials

## Abstract

We prepared FeCoPt/SiO_2_ thin films by sol-gel spin-coating technique. As-prepared composite films were reduced in hydrogen to induce texture growth. Structural, magnetic property and surface morphology of the films were characterized by X-ray diffraction (XRD), vibrating sample magnetometer (VSM) and scanning electron microscope (SEM). These experimental data indicate that integrated intensity ratio *I*_(200)_/*I*_(110)_ of diffraction peaks (200) and (110) of FeCo firstly increases and then decreases, while the coercivity first decreases and then increases with increasing Pt doping content. The specimen with less Pt doping content has a large *I*_(200)_/*I*_(110)_ value and small coervicity value, which is closely related with strong (200) texture in FeCo thin film. These results indicate that fcc-Pt is also in favor of promoting (200) FeCo texture like Al or Cu elements, and this similar trends of Pt and Al originate from their similar atomic radius and crystal cell volume.

## Introduction

Compared with traditional materials, nanomaterials have received considerable attention due to their unique physical and chemical properties^1–7^. Magnetic nanomaterials have wide applications in the fabrication of optical and electronic devices, since their remarkable magnetic properties can improve the performance of those devices, for examples in power generation, conditioning, conversion, transportation, and other energy-use sectors of the economy^8–13^. Nanometer-sized FeCo alloys have been paid enough attention by many researchers recently since they possess much better soft magnetism such as low *H*_c_ (coercivity), high *μ* (permeability), hig h *T*_c_ (Curie temperature), high *M*_s_ (saturation magnetization) and high tensile strength^14–17^. Due to the formation of the ordered B2 state^18^, near equiatomic FeCo alloys exhibit very good soft magnetic properties, while they are extremely brittle at room temperature^19^, on the other hand, the coercivity of FeCo alloy films prepared by a conventional sputtering method is quite high, which is naturally not applicable for high frequency application^20^. So a suitable non-magnetic matrix and a strong (200) FeCo texture are vital on improving FeCo soft magnetism and further applications. Our teams have devoted some years to investigating how to improve (200) FeCo texture growth^21^ by choosing different metals as seedlayer or underlayer (Cu^22^, Co^23^ and Al^24^) and doping elements (Al^25^, Cu, Pt and Pd) into FeCo crystal lattice and also by choosing various preparing methods. Sol-gel technique is not only commonly easy-control to achieve uniform-doping among raw materials but also combines a superior advantage of low-cost.

In this work we chose Pt metal as doping element in view of the tiny structural difference between fcc-Pt, fcc-Al, fcc-Cu and bcc-FeCo, for the purpose to investigate the influence of deferent elements on FeCo grains’ preferred orientation.

## Experiments

Cobalt nitrate (Co(NO_3_)_2_·6H_2_O), platinum nitrate (Pt(NO_3_)_2_) and iron nitrate (Fe(NO_3_)_3_·9H_2_O) were dissolved into absolute ethyl alcohol under continuously magnetic stirring, then some mixed solution of glycol and ethyl orthosilicate were dropwise-added into as-prepared initial solution, then some nitrate was added to get transparent sol. The sol was ultrasonic vibrated for 1 hour and aged at room temperature for 24 hours, then spin-coated onto silicon substrates, as-prepared films were annealed in hydrogen for 1 hour to obtain FeCoPt/SiO_2_ thin films. The Pt doping content was set as 0, 0.5, 1, 3 and 5 wt% in FeCo/SiO_2_ composite films, the corresponding films were marked as a, b, c, d and e, respectively.

A Rigaku D/max 2500/PC X-ray diffraction was chosen to investigate the structures of the samples, and radiation is Cu K_*α*_. Lake Shore M-7407 vibrating sample magnetometer was chosen to study the magnetic properties of the specimens, the films were measured at room temperature. A JSM-7800F field emission scanning electron microscope was used to observe the surface topography of film samples.

## Results and Discussion

**Figure 1:**
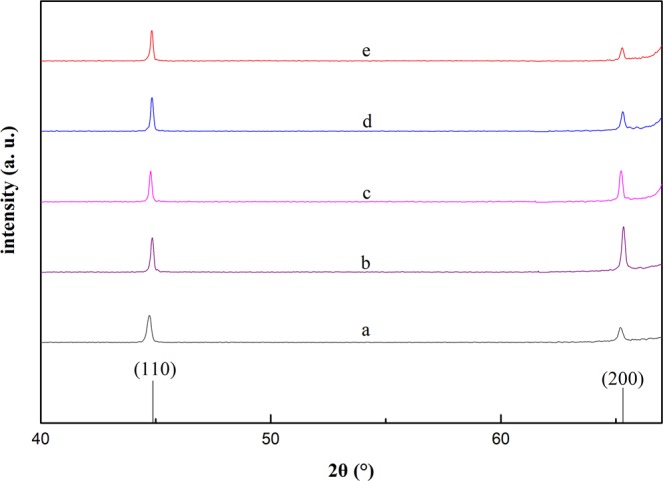
XRD patterns of the samples with various Pt doping content.

XRD patterns of FeCoPt/SiO_2_ sample films were shown in Fig. 1. There were only two diffracting peaks which can be indexed to (110) and (200) planes of bcc structure FeCo alloy. No diffracting peak corresponds to silicon dioxide. This indicates that silicon dioxide is amorphous. The diffracting peaks corresponding to metal platinum could not be detected also, which is due to the replacement of Fe or Co sites by Pt elements, since Pt is doped into FeCo alloy crystal lattices. As seen in Fig. 1, the intensity of (200) peak for the sample b is higher than that of (110) peak. This indicates that the film tends to present (200) preferred orientation after being doped with only small amount Pt element (0.5 wt %). But this preferred orientation trends becomes less obvious with the increasing of Pt doping, since the peak intensity of FeCo (200) becomes small gradually when the Pt doping content increases.

**Figure 2:**
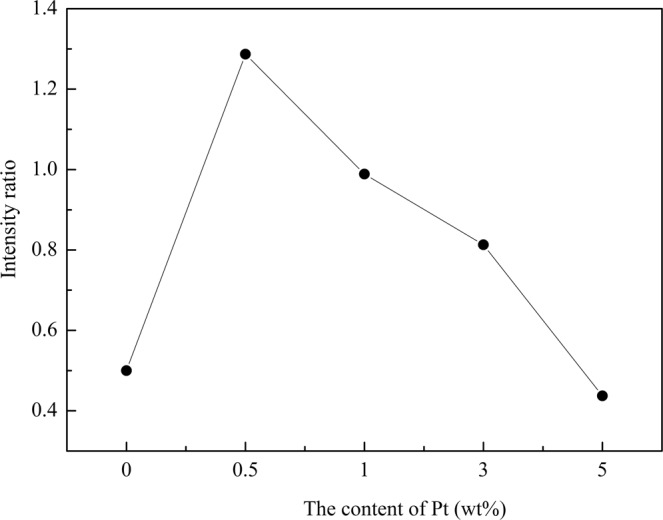
Dependence of the *I*_(200)_/*I*_(110)_ versus various Pt doping content.

We calculated the integrated intensity ratio of diffraction peaks (200) to (110), which is noted as *I*_(200)_/*I*_(110)_. In Fig. 2, we presents the relation between the *I*_(200)_/*I*_(110)_ and Pt doping content. It displays that the value of *I*_(200)_/*I*_(110)_ reduces when Pt doping content increases. The maximum value of *I*_(200)_/*I*_(110)_ appears when Pt doping is 0.5 wt%. This indicates that a strong (200) texture of FeCo arises on the smallest Pt doping. Samples with Pt doping follow the same trend as Al element both as doping and underlayer^24,25^. It is obvious that both metal Al and Pt can promote FeCo (200) preferred orientation, but this promotion was reduced when more Al or Pt were doped into or deposited. While for samples underlayered with Cu and Co^22,23^, they present a different trend. For example, the more is the thickness of Co, the higher is the value of *I*_(200)_/*I*_(110)_, which means more Co content is benefit for improving FeCo (200) preferred orientation. This various doping effect is dominantly derived from their structural and crystal difference between those metals. It is due to the similar atomic radius and crystal cell volume values between Al and Pt, so they can result in similar effects among FeCo grains. The corresponding parameters are listed in Table 1.

**Table 1:** Element parameters of Fe, Al, Cu and Pt metals.

Elements	Atomic radius (Å)	Electronegativity	Crystal structure	Volume (cm^3^/mol)	Radius difference with Fe
Fe	1.56	1.8	BCC	7.1	0
Cu	1.57	1.9	FCC	7.1	0.01
Al	1.82	1.5	FCC	10	0.26
Pt	1.83	2.2	FCC	9.1	0.27
Co	1.67	1.9	Hexagon	6.7	0.11

For Pt element, it has similar atomic radius and crystal cell volume with Al element, so they play similar role in doping into FeCo alloys’ crystal lattices, thus cause similar relative lattice strain between FeCo grains, resulting in similar relative lattice deformation (*d*-*d*_0_)/*d*_0_ values, which is dominant in promoting preferred orientation in (200) planes or (110) planes^26^. It is also noted that the more element doping whether Al or Pt are introduced, the more relative lattice strain are formed, so appropriate choosing of doping element and doping content is important in promoting (200) texture in FeCo thin films. For the samples underlayered with Cu or Co, they display different changing trend due to their structural and crystal difference from Al and Pt.

**Figure 3:**
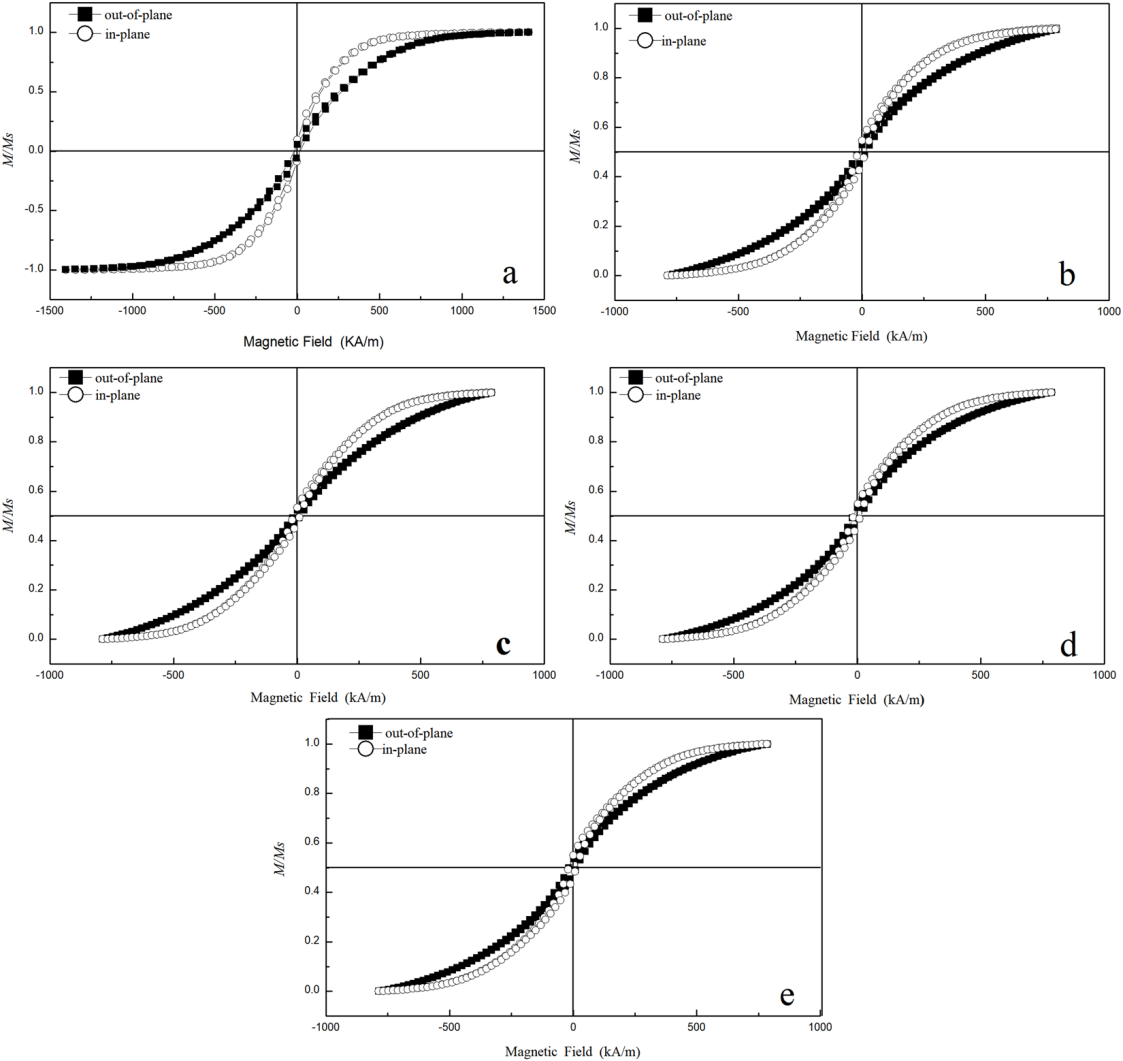
The hysteresis loops of the samples with various Pt doping content.

The magnetic hysteresis loops of the FeCoPt/SiO_2_ sample films are shown in Fig. 3, and it shows that all samples films display very typical soft magnetism. Corresponding magnetic data of Pt, Al, Cu and Co metals were listed in Table 2.

**Table 2:** Magnetic data of Pt, Al, Cu and Co metals.

doping	Element	Content/(wt%)	0	0.5	1	3	5
	Pt	*Hc*_*∥*_/(kA/m)	15.01	14.44	12.21	13.88	15.09
		*Hc*_⊥_/(kA/m)	17.3	15.25	13.26	14.49	15.39
	Al	*Hc*_*∥*_/(kA/m)	15.01	12.03	11.96	14.14	19.06
		*Hc*_⊥_/(kA/m)	17.3	14.09	15.05	16.83	21.01
underlayer	Element	*Thickness*/(nm)	1	2	3	5	10
	Al	Hc_*∥*_/(kA/m)	5.50	5.35	5.57	5.70	5.46
		Hc_⊥_/(kA/m)	5.70	6.02	2.53	6.25	5.49
	Cu	Hc_*∥*_/(kA/m)	3.28	3.15	3.34	3.36	3.35
		Hc_⊥_/(kA/m)	4.57	4.25	4.50	4.78	4.61
	Co	Hc_*∥*_/(kA/m)	6.10	5.57	5.48	5.61	5.20
		Hc_⊥_/(kA/m)	5.78	5.30	5.34	5.02	5.11

**Figure 4:**
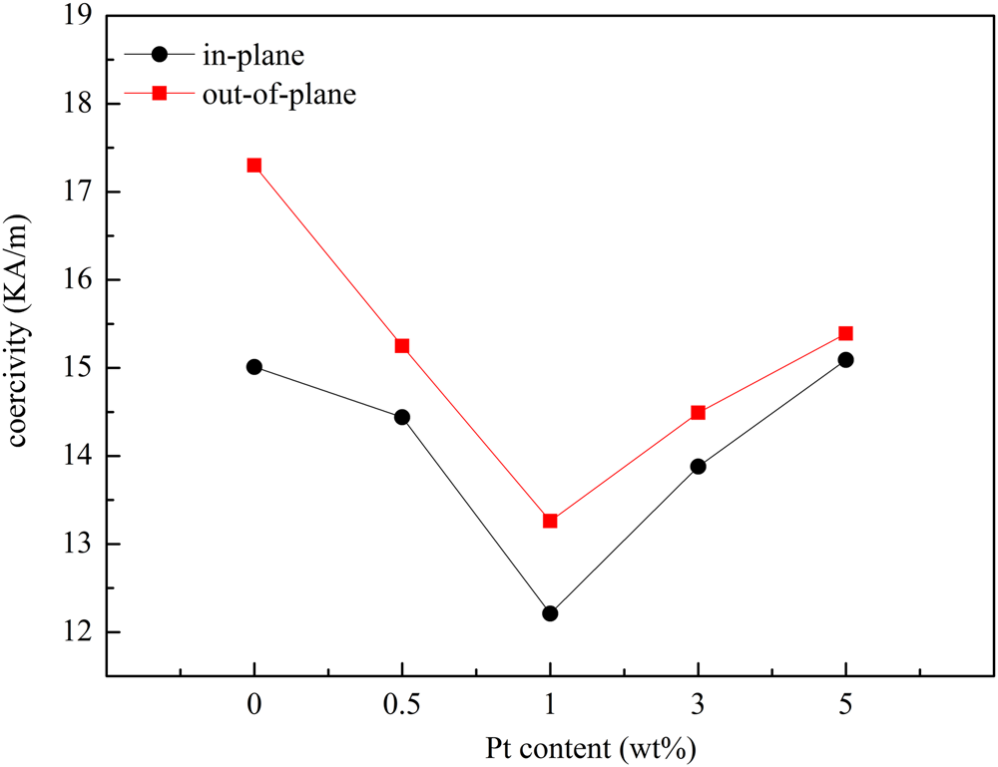
Dependence of coercivity *H*_c_ of the samples versus Pt doping content.

Figure 4 illustrates the graphs of the values of the coercivity *H*_c_ of the films. As shown in Fig. 4, all the *H*_c ⊥_ (out-of-plane) is higher than *H*_c*‖*_ (in-plane), this shows that an out-of-plane magnetic anisotropy is dominating for all sample films. It can be shown from previous XRD results, there exists the smallest strain between FeCoPt/SiO_2_ film so presenting a strong (200) texture when Pt doping content is less than 1 wt%, this leads to expressing the best soft magnetic property with less coercivity *H*_c_ values. So Pt is appropriate to be chosen as doping element for improving FeCo (200) texture growth as Al element.

This kind of similar atomic and crystal structure between Pt and Al elements produce similar relative lattice strain between FeCo grains when they act with FeCo particles whether as doping elements into FeCo crystal lattice or as underlayers being deposited under FeCo layers, so they play similar role in promoting (200) texture in FeCo thin films, which is also the key factor in governing their magnetic properties. For samples underlayered with Co or Co element, whether the values of coercivity in-plane and out-of-plane, or the changing trends follow the different ways, originating from their structural and crystal difference with Al and Pt elements.

**Figure 5:**
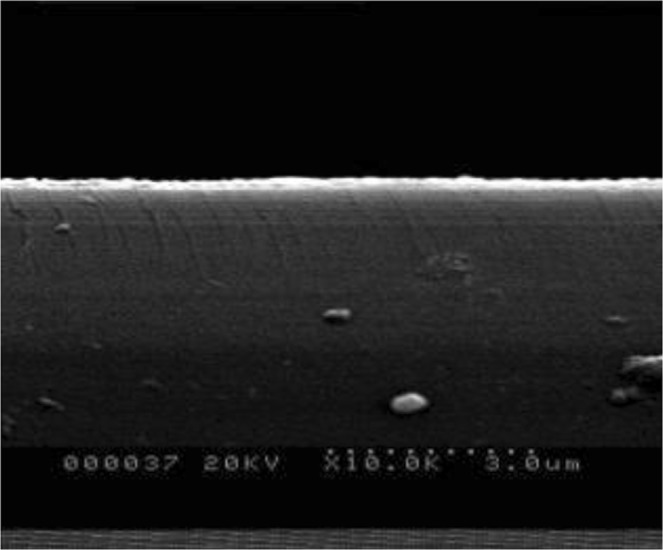
SEM images of sample.

Figure 5 shows the SEM images of sample film. The thickness of the sample film is calculated as 110 nm by Nano Measure software and the grains’ growth is homogeneous.

## Conclusions

Pt metal is proved to be a good doping element as Al is introduced into FeCo alloys to promote (200) preferred orientation. It is due to similar atomic radius and crystal cell volume of Pt and Al that the change of structural and magnetic properties of FeCoPt/SiO_2_ thin films follows the similar trends with the increasing of Pt doping content.

## Data Availability

The data used to support the findings of this study are included within the article.
